# Giant mass but small symptoms; huge thrombosis in the right atrium originating from the superior vena cava and protruding to the right ventricle: a case report

**DOI:** 10.1186/s13256-019-2240-3

**Published:** 2019-10-19

**Authors:** Elnaz Javanshir, Seyyed-Reza Sadat-Ebrahimi, Rezayat Parvizi, Mehrnoosh Toufan, Hosein Sate

**Affiliations:** 1 Cardiovascular Research Center, Tabriz University of Medical Sciences, Tabriz, Iran; 2 Student Research Committee, Tabriz University of Medical Sciences, Tabriz, Iran

**Keywords:** Superior vena cava, Thrombosis, Right atrium mass, Pulmonary thromboembolism

## Abstract

**Background:**

Thrombosis of the superior vena cava with propagation to the right heart chambers can be seen in the presence of chronic indwelling catheters. Moreover, the idiopathic right atrial thrombi may become entrapped in Chiari’s networks, and idiopathic thrombosis of the superior vena cava may occur rarely because of the underlying coagulation disorders or malignancies.

**Case presentation:**

A 43-year-old Iranian (Persian) woman was admitted to our hospital with palpitation of 2 years’ duration and mild to moderate dyspnea of 10 days’ duration. Her past medical history, basic laboratory test results, and cardiac enzyme measurements were unremarkable. Imaging studies revealed a 1.4-cm × 7.4-cm multilobulated, hypermobile mass in the right atrium, extending into the right ventricle, that appeared to be emanating from the superior vena cava. Moreover, partial filling defects were visible in the distal parts of both right and left pulmonary arteries extending to their branches, suggesting massive pulmonary emboli. The patient’s huge mass and emboli were removed by surgery, and pathologic evaluations confirmed that all of the specimens were thrombosis. A number of mutations known as risk factors of thrombosis were detected during genetic evaluations. However, mild symptoms of the patient along with a huge mass in the right atrium, thrombosis in the superior vena cava, and massive thromboembolism remained unexplained.

**Conclusion:**

Huge and dangerous thrombosis inside the heart and superior vena cava can evolve without expected considerable symptoms. Also, detecting the underlying causes of these thromboses sometimes is not feasible by only checking the prevalent known risk factors. Therefore, comprehensive evaluations should be carried out in these patients.

## Background

Superior vena cava (SVC) thrombosis is a rare phenomenon that can develop from venous access devices implantation [1] or coagulopathies such as protein S deficiency [2], Behçet disease [3], or malignancies. In the present report, we describe an unusual case of huge thrombosis originating from the SVC, extending into the right atrium (RA), and protruding to the right ventricle (RV) with massive thromboembolism. None of the aforementioned etiologies were perceived in our patient. Moreover, huge masses in the RA and massive thromboembolism are supposed to bring about severe symptoms, but our patient only showed a mild to moderate dyspnea. This case is reported with permission granted by our institutional review board (IRB).

Two major issues persuaded us to report this case:

1. Although symptoms are not always directly associated with the underlying disorders in a variety of diseases, some disorders are supposed to bring about considerably dramatic symptoms, such as massive pulmonary thromboembolism or development of a giant mass inside the chambers of the heart. Consequently, rigorous interventions and critical care might be ignored in those patients with catastrophic pathologies demonstrating mild symptoms.
2. The occurrence of thrombosis is not a rare finding; however, there are very few reports of development of thrombosis in some sites, such as the SVC, especially in the absence of iatrogenic causes such as venous access device implantation. Therefore, detecting thrombosis in those sites requires a more comprehensive evaluation. In our patient, we found some genetic mutations predisposing her to thrombosis.

## Case presentation

A 43-year-old Iranian (Persian) woman was admitted to the Shahid Madani Hospital with palpitations and dyspnea. She had been experiencing palpitations for 2 years and recently (since about 10 days before admission) had developed dyspnea with New York Heart Association functional class II. Her past medical history was unremarkable, including usual childhood illnesses and no history of rheumatic fever. She never experienced atrial fibrillation and was receiving a low dosage of propranolol (10-mg tablet daily), fluoxetine (10-mg capsule daily), and alprazolam (0.5-mg tablet daily) for her palpitations. She was not receiving oral contraceptive medications. She was the mother of two healthy children, who were both delivered through normal vaginal delivery. She did not have any history of abortion. Her familial history was also unremarkable. She was a housewife who lived with her husband and her two children, never had a job, and was financially supported by her husband. She had never smoked or consumed alcohol or opiates, and she did not follow any special diet.

At the time of admission, her vital signs were within normal ranges (blood pressure 110/70 mmHg, pulse rate 95 beats/minute, respiratory rate 23/minute, oxygen saturation 95%), and her physical examinations revealed nothing considerable. Her extraocular motions full, her gross visual fields were full to confrontation, and her conjunctiva were clear. Her scleras were nonicteric, her pupils equal round and reactive to light and accommodation, and her fundi were also normal. Her hearing was normal. Her tympanic membrane landmark was well visualized. Pharyngeal injection with exudates was not noted. Her uvula moved up in midline. She had a normal gag reflex. Her jugular venous pressure was 8 cm, and her thyroid was not palpable. She had no masses and no adenopathy. Her lung auscultation was normal. She had no rales, rhonchi, wheezes, or rubs. No dullness to percussion was detected. Her diaphragm moved well with respiration. She had no heaves or thrills. She had a normal S1 heart sound, and her S2 sound was narrowly split. No murmur or abnormal heart sound was detected by auscultation. Her pulses were notable for sharp carotid upstrokes. Distal pulses of her extremities were 2+ and symmetric. Her neurologic examination revealed that she was awake, alert, and fully oriented. Her cranial nerves I–XII were intact. Her strength was normal. Her sensory examination revealed normal responses to touch and pinprick. No edema of her arms or head was noticeable. She had no tremor or dysmetria.

Her basic laboratory test results and cardiac enzyme findings were normal, except for mild leukocytosis (white blood cell count 14,400), which remained in the same range until discharge. The results of some other laboratory tests were as follows: hemoglobin, 12.5 g/dl; hematocrit, 41.0%; mean corpuscular volume, 89.0 fl; platelets, 256,000; reticulocyte index, 1.6%; Na^+^, 140 mEq/L; K^+^, 4.2 mEq/L; blood glucose, 102 mg/dl; blood urea nitrogen, 19 mg/dl; serum creatinine 1.0 mg/dl; total bilirubin, 0.5 mg/dl; direct bilirubin, 0.1 mg/dl; alkaline phosphatase, 135 mg/dl; aspartate aminotransferase, 45 IU/L; alanine aminotransferase, 27 IU/L; lactate dehydrogenase, 413 IU/L; erythrocyte sedimentation rate in first hour, 27 mm; creatine phosphokinase, 21 IU/L; creatine kinase-MB, 12%; cardiac troponin I, 0.1 ng/ml; hepatitis B surface antigen, negative; C-reactive protein, negative; and rheumatoid factor, negative. Urine analysis revealed specific gravity, 1.003; pH, 6.5; protein, negative; blood, negative; white blood cell count, 2–3; red blood cell count, negative; and bacteria, negative. Arterial blood gas tests revealed pH, 7.43; pCO_2_, 38 mmHg; PO_2_, 88 mmHg; HCO_3_^−^, 23 mEq/L; and oxygen saturation, 98% on room air.

**Fig. 1:**
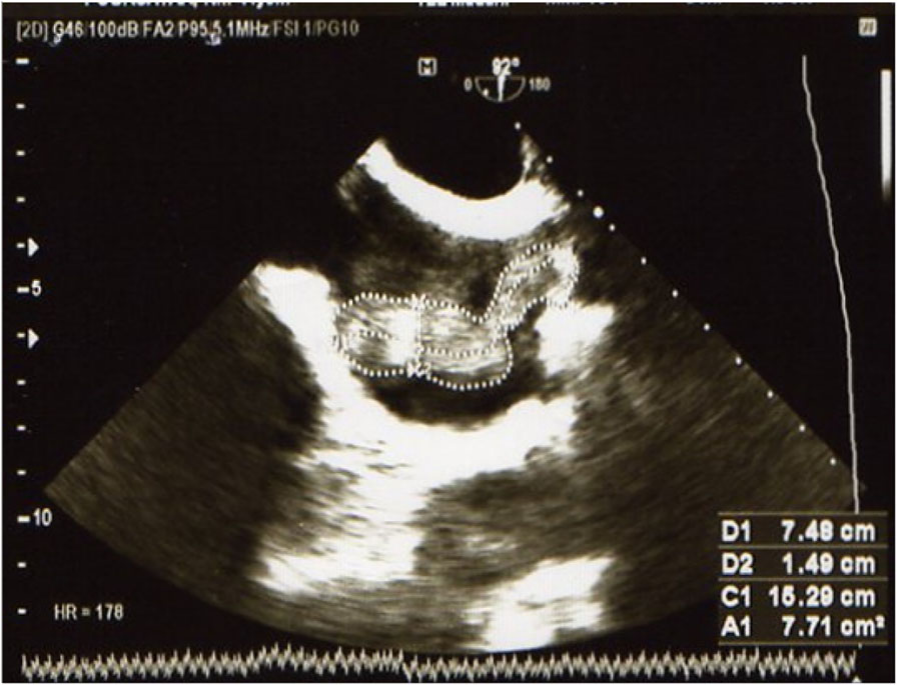
Bicaval view in transesophageal echocardiography showing a huge multilobulated mass in the right atrium and involvement of the upper part of the superior vena cava

Electrocardiography demonstrated tachycardia (rate about 100 beats/minute) without any cardiac arrhythmia. No sign of RV overload was noticeable. Primary transthoracic echocardiography revealed a mass in the RA; therefore, transesophageal echocardiography (TEE) was performed. TEE revealed RA enlargement and a huge, dense, multilobulated, hypermobile mass in the RA (1.4 cm × 7.4 cm) and extending into the right RV, which appeared to be emanating from the SVC (Fig. 1). Furthermore, the mass was noted in the upper part of the SVC (20 mm from the mouth); however, the left ventricle (LV) and right ventricle (RV) had normal size, and their normal systolic functions were preserved (LV ejection fraction, 55%). Although the RA area was enlarged (23cm^2^), its systolic pressure was 43 mmHg, displaying moderate pulmonary hypertension. The main pulmonary artery and its branches were also dilated. The valvular evaluation demonstrated mild mitral regurgitation and mild tricuspid regurgitation, but no septal defect or patent foramen ovale was seen.

**Fig. 2:**
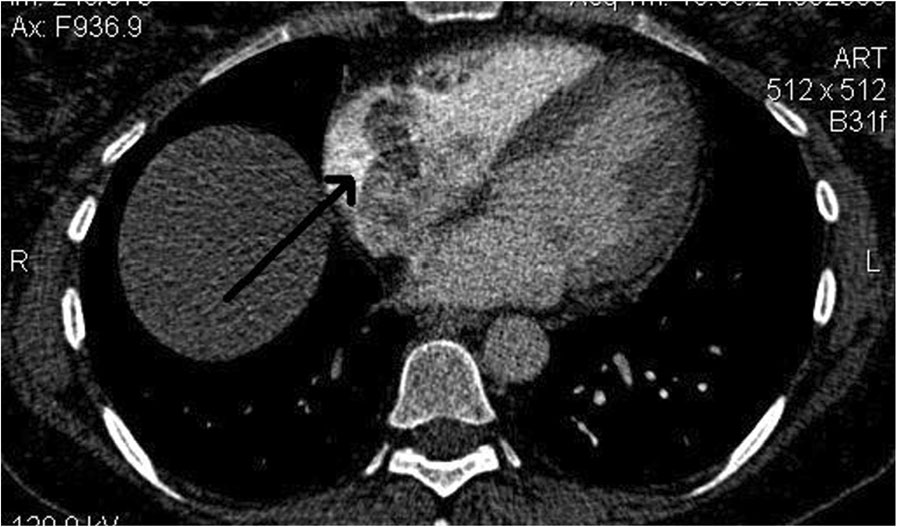
Computed tomographic angiography demonstrating the hypodense filling defect in the right atrium. The arrow is pointing to this filing defect

**Fig. 3:**
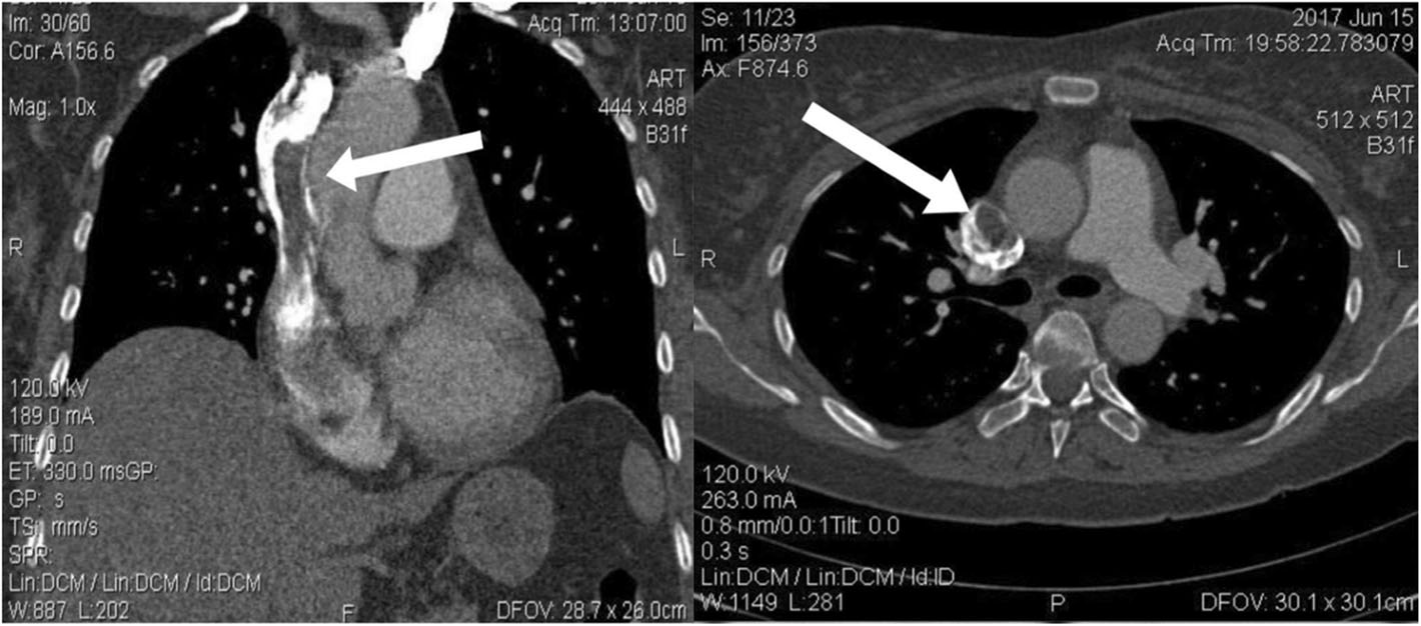
Computed tomographic angiography demonstrating a filling defect in the superior vena cava. The arrow is pointing to these filing defects

**Fig. 4:**
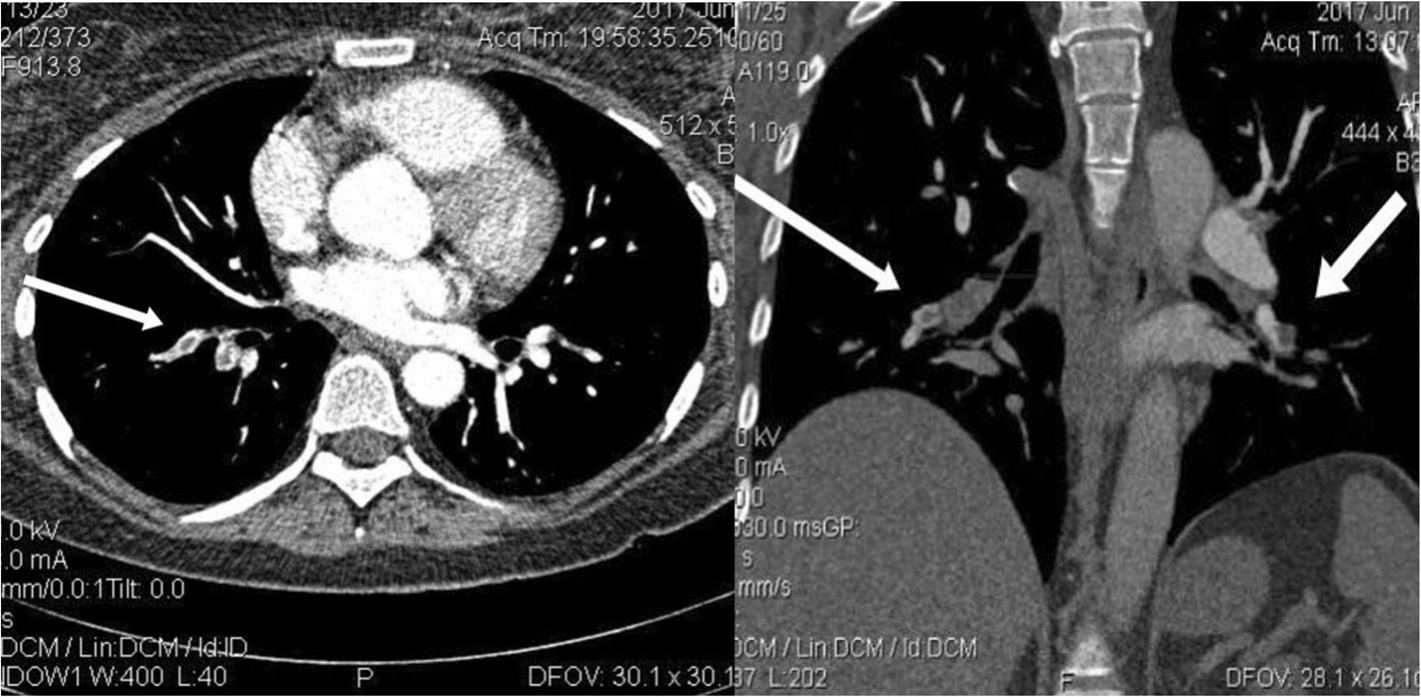
Computed tomographic angiography demonstrating filling defects in segmental right and left pulmonary arteries. The arrow is pointing to these filing defects

Systemic anticoagulation was immediately instituted with intravenous heparin, and the patient was transferred to the cardiac intensive care unit. Likewise, computed tomographic (CT) angiography demonstrated a partial filling defect in the SVC that was connected to another filling defect in the RA (46 mm × 26 mm) (Figs. 2 and 3). Moreover, partial filling defects were visible in the distal part of the right and left pulmonary arteries, extending to their branches, proposing massive pulmonary emboli (Fig. 4). Atelectatic bundles were seen in the middle and lingual lobes, but the size of the heart was normal, and no pleural effusions were detectable.

The contrast-enhanced CT scans and ultrasonographic findings for the chest and abdomen did not show any evidence of tumor or malignancy. Also, there was no evidence of venous filling defects below the diaphragm or deep vein thrombosis (DVT) of the lower extremities. Because of the hypermobile huge mass in the RA and following a cardiac surgeon’s consultation, the patient was indicated for surgery and later was prepared for cardiac surgery. Anticoagulation therapy was continued until the patient was transferred to the operating room. Coagulation test results prior to the surgery were prothrombin time 12 seconds (international normalized ratio [INR] 1) and partial thromboplastin time 60 seconds.

**Fig. 5:**
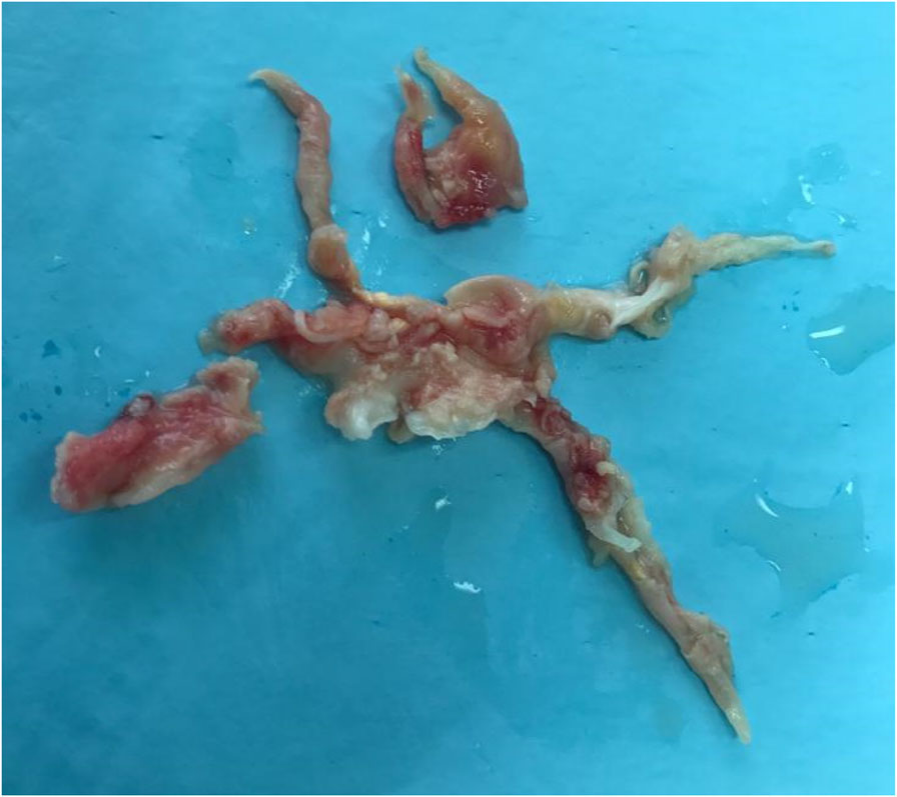
The extracted clots from the pulmonary arteries

At the time of surgery, following a median sternotomy, cardiopulmonary bypass (CPB) was instituted following cannulation of the ascending aorta and the inferior vena cava. The RA and SVC were opened, and the long-standing “white clots” were extracted. Afterward, the heart was arrested with St. Thomas’ Hospital cardioplegic solution, and circulatory arrest with antegrade cerebral perfusion was initiated. Clots were also removed from the branches of both the right and left pulmonary arteries (Fig. 5). All the suture lines were closed, and the patient was weaned from CPB following 152 minutes with cross-clamping and circulatory arrest times of 112 and 20 minutes, respectively. Intraoperative TEE after clot removal revealed normal LV size and systolic function, and there were not any signs of clots in the SVC, RA, or other parts. The patient tolerated the procedure well and had an uncomplicated postoperative course. The final TTE did not show any signs of recurrence of the masses or pulmonary hypertension. The pathologic evaluations confirmed that all the surgically removed specimens were thromboses. A complete coagulation workup revealed heterozygosity in the following genes: factor V Leiden HR2 (4070 A/G), methylenetetrahydrofolate reductase (MTHFR) (1298 A/C), β-fibrinogen (− 455 G/A), glycoprotein Ia (807 C/A), plasminogen activator inhibitor 1 (PAI-1; 5G/4G), tissue plasminogen activator (intron 8 del/ins), and apolipoprotein E (E2/E3).

The patient’s symptoms, including palpitation and dyspnea, dissolved completely after the surgery, and she was discharged on warfarin to maintain an INR of 2–3. Finally, she was followed for 1 year after her discharge for any symptoms and signs of the clots, but the follow-up results were all negative. No pulmonary hypertension was detected by TTE in follow-up.

## Discussion

We report an unusual case of a patient with massive thrombosis originating from the SVC with extension into the RA and protrusion to the RV with mild symptoms. She was admitted to our hospital with palpitation, mild to moderate dyspnea, and tachycardia without and specific abnormality in her physical examinations; however, evaluations by echocardiography and CT angiography revealed a huge mass in her cardiac chambers and SVC as well as massive thromboembolism. The pathologic evaluations confirmed that all specimens were thromboses. Further genetic workup revealed heterozygosity in some genes that were predisposing factors for thrombosis.

Three dimensions of this rare case of thromboembolism need to be discussed: first, the SVC thrombosis, which is a rare site of thrombosis, especially in cases with no previous interventions; second, the appearance of an RA mass; and third, mild symptoms of a massive pulmonary embolism (PE). The occurrence of thrombosis in such an unusual site drove us to reconsider the possible disorders of basic parameters that have interplay in developing venous thrombosis, including primary hypercoagulation defects in proteins governing coagulation and fibrinolysis or secondary hypercoagulable states or disorders involving blood vessels and blood flow or stasis [4]. It has been indicated that the most common cause of the hypercoagulable state is the antiphospholipid syndrome, followed by factor V Leiden (1691 G/A) mutation, prothrombin gene G20210A mutation, elevated factor VIII, and hyperhomocysteinemia, Less common disorders are deficiencies in antithrombin, protein C, or protein S [5]. Acquired causes of venous thrombosis have been suggested as surgery; malignancy; and, less commonly, trauma, pregnancy, long-haul travel, obesity, oral contraceptive use, hormone replacement therapy, myeloproliferative disorders, and polycythemia vera [4]. Although some special cases of SVC thrombosis were previously reported with a known cause, including protein S deficiency [2], Behçet disease [3], or malignancies, our patient did not have any of them on the basis of meticulous evaluations. Moreover, owing to the special location of the SVC, stasis could not be considered. We hypothesize that the presence of her genetic mutations increased her risk of thrombosis. Mutations in factor V Leiden HR2 (4070 A/G) and glycoprotein Ia genes (807 C/A) are associated with an increased risk of developing thrombosis [6, 7]. Also, the mutation in the MTHFR gene (1298 A/C), when it is present together with heterozygosity in C677T, is associated with a reduction in MTHFR enzymatic activity; therefore, these patients are predisposed to mild hyperhomocysteinemia and consequently hypercoagulable states [8]. It has been suggested that mutation in the β-fibrinogen gene (− 455 G/A) induces a higher level of plasma fibrinogen [9], which is related to a higher risk of thromboembolism [10]. Hypofibrinolysis due to excess of PAI-1 is associated with DVT. In addition, 4G/5G polymorphism of the PAI-1 gene may modify the inhibitor’s synthesis [11], and it has been postulated that the mutation of apolipoprotein E gene (E2/E3) is associated with coronary artery disease [12].

Regarding the fact that RA masses are quite rare findings, their possible etiologies are mostly myxoma, metastasis of extracardiac malignant tumors such as renal cancer, intravenous leiomyomatosis, thrombosis, vegetations, and normal variants [13]. A definitive diagnosis cannot be made without histological examinations. According to the pathologic evaluations of our patient, the detected primary mass in the RA was a thrombosis. The sources of the thrombi in the RA are mostly the deep veins, and when the embolization occurs, it entraps in the tricuspid valve or in the RV trabeculations during their intracardiac transit. Thrombi can also be caused by indwelling catheters or pacer wires [14]. None of the mentioned causes were detected in the history of our patient; the only possible cause was imagined to be the hypercoagulable state due to her genetic mutations.

The third important concern of this patient was her mild symptoms despite massive pulmonary artery occlusion. Acute PE causes symptoms and signs immediately after the obstruction of arteries, but patients with chronic PE mostly develop slowly progressive dyspnea over a period of years due to pulmonary hypertension. The chronic PE commonly occurs in patients with a history of acute PE and etiologies similar to those mentioned for SVC thrombosis. Despite the moderate pulmonary hypertension of our patient and the white appearance of her clots, which indicated their chronic presence, the dyspnea of the patient had appeared only recently. Moreover, the patient’s palpitation was most probably due to the huge mass in the RA, because it was previously reported in other similar cases [13, 15].

## Conclusion

A huge and dangerous thrombosis inside the heart and the SVC can evolve without expected considerable symptoms; therefore, the prevalent known risk factors in diagnosing these patients should not always be expected to be found, and comprehensive evaluations should be carried out in patients similar to ours.

## Data Availability

All data and material collected during this study are available from the corresponding author upon reasonable request.
